# Optic Disc Segmentation by Balloon Snake with Texture from Color Fundus Image

**DOI:** 10.1155/2015/528626

**Published:** 2015-03-16

**Authors:** Jinyang Sun, Fangjun Luan, Hanhui Wu

**Affiliations:** ^1^School of Information & Control Engineering, Shenyang Jianzhu University, Shenyang 110168, China; ^2^Sino-Dutch Biomedical and Information Engineering School, Northeastern University, Shenyang 110819, China

## Abstract

A well-established method for diagnosis of glaucoma is the examination of the optic nerve head based on fundus image as glaucomatous patients tend to have larger cup-to-disc ratios. The difficulty of optic segmentation is due to the fuzzy boundaries and peripapillary atrophy (PPA). In this paper a novel method for optic nerve head segmentation is proposed. It uses template matching to find the region of interest (ROI). The method of vessel erasing in the ROI is based on PDE inpainting which will make the boundary smoother. A novel optic disc segmentation approach using image texture is explored in this paper. A cluster method based on image texture is employed before the optic disc segmentation step to remove the edge noise such as cup boundary and vessels. We replace image force in the snake with image texture and the initial contour of the balloon snake is inside the optic disc to avoid the PPA. The experimental results show the superior performance of the proposed method when compared to some traditional segmentation approaches. An average segmentation dice coefficient of 94% has been obtained.

## 1. Introduction

Glaucoma is the second most common cause of blindness worldwide [1]. It is also the leading cause of blindness among African Americans. There will be 60.5 million people with open angle glaucoma (OAG) and angle closure glaucoma (ACG) in 2010; the number will be increasing to 79.6 million by 2020 [2]. An automatic and economic system is highly desirable for glaucoma screening for a wider range of people. The best approach to do screening is based on 2D color fundus image as it is cheap and easy to manipulate. The optic nerve head assessment based on the 2D color fundus image is the best way for glaucoma screening at the moment. But automatic glaucoma screening is based on the risk factors from the optic nerve head, so an accurate segmentation of optic nerve head is the basic and most important process in glaucoma screening.

**Figure 1:**
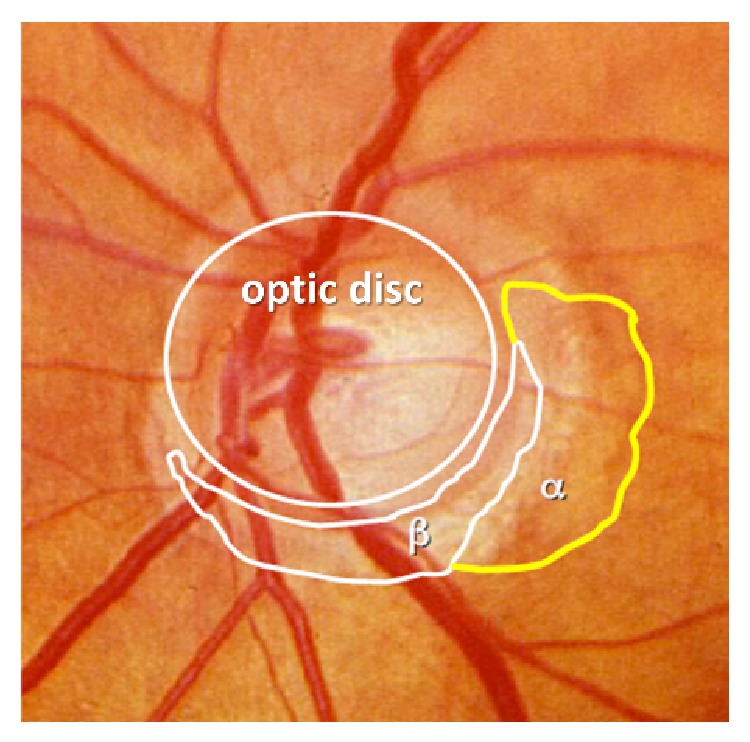
Color fundus image. The Zone alpha and beta peripapillary atrophy is near the disc.

In retinal images, the optic disc generally appears as bright, yellowish, circular or slightly oval-shaped object as shown in Figure 1.

Optic disc segmentation can be generally grouped into three categories based on the methods for extracting the optic disc boundary:

- template matching or Hough transform methods,
- deformable models like snake, level sets,
- supervised classification or unsupervised classification.

Shape-based template matching [3, 4] is a very easy approach to segment the disc as the optic disc is approximately circular or elliptical. But this method is not very accurate as the shape of the optic disc is not a perfect circle or ellipse due to some pathological changes and this method often fails as there is PPA near the optic disc. Mohammad et al. [5] use binary robust independent elementary features (BRIEF) and a rotation invariant BRIEF (OBRIEF) features to find approximated boundary of the optic disc. Then a pixel classification method followed by circular template matching to segment the optic disc.

Active contour model is one of the most promising approaches for OD segmentation as it is better in capturing irregularity disc region. GVF-snake algorithm is proposed by Osareh et al. [6] to extract the optic disc boundary. In order to remove the vessel occlusion during the deformable model, the blood vessel was first removed by morphology in the preprocessing step. Walter et al. [7] also use morphological filtering techniques to remove the blood vessels and they detect the optic disc boundary by means of watershed transformation.

Li and Chutatape [8] used principal component analysis (PCA) to locate the disc first and applied a modified active shape model (ASM) for optic disc identification. By means of shape model, this approach can handle vessel occlusion and fuzzy edges. However, the shape of the optic disc boundary may have different pathological changes. Hence, these template restrictions may reduce the accuracy of the segmentation. Lowell et al. [9] use global elliptic model to estimate the optic disc location and radius; then they apply a local deformable model with variable edge-strength dependent stiffness. This algorithm often fails where there are large numbers of white lesions, PPA, or strongly visible choroid vessel [10]. In order to solve the vessel occlusion and fuzzy contour shapes, Xu et al. [10] propose a knowledge-based clustering and smoothing update deforming model. After each deformation, the contour points are classified into two clusters by knowledge-based unsupervised learning. Then the contour is updated using both global and local information, so this method can achieve balance on contour stability and accuracy. They showed their proposed method achieves better success rate (94%) when comparing to GVF-snake (12%) and modified ASM (82%) approach. However, they also show that this method fails where there is obvious white PPA region near the disc. Dashtbozorg et al. [11] propose an automatic approach for OD segmentation using a multiresolution sliding band filter (SBF) and the OD boundary is regularized using a smoothing algorithm. This approach gets an average overlapping area of 83%, 89%, and 85% in three datasets.

All of these deformation methods are very sensitive to the initial contour. More recently, region-based active contour model is presented in [13]; this method can detect the fuzzy edge and is not sensitive to initial contour. However, the Chan-Vese model cannot handle inhomogeneous image which may lead to erroneous segmentations. Joshi et al. [14] propose a new segmentation method based on localized CV models [15] using local image information from three-dimensional feature spaces. This is a novel method by using texture feature to segment the optic disc, and they show a very nice result in their paper. But the local Chan-Vese active contour model can lead to oversegmentation as the texture of the retinal image is inhomogeneous. The selection of local neighbor radius parameter which defines the local image domain around a point of interest is very important. If the radius is too small this model is just like GVF model, while a large radius will decrease the sensitivity to small gradients and make this model be similar to the traditional Chan-Vese model and PPA region will be misclassified to optic disc region.

Another way to segment the optic disc is based on pixel classification [16, 17]. The difficulty of this approach is the shape of the classification result is often unsmooth. Moreover, it is hard to find a good texture feature to distinguish the PPA and optic disc. And this approach also needs to be trained before being applied to classification and it is very time consuming to segment the optic disc by SVM when exploiting lots of features for learning.

The difficulty in optic disc segmentation is caused by PPA as shown in Figure 1. The traditional methods often mistake PPA region as a part of optic disc. In order to solve the problem, we use the texture information from image which is robust to image inhomogeneity. This method is based on a balloon snake model, unlike other active contour models, such as snake or level set, which use gradient information to make the contour stop at the edge of disc. Thus, those methods require the initial contour to be near the true optic disc boundary. In this paper, we apply a fuzzy *c* means method to exclude those noises and keep the edge texture near the boundary. Then we choose the image texture rather than gradient to do segmentation, and the initial contour of the snake is a small circle in the disc region to avoid PPA region.

The organization of this paper is as follows. In Section 2, we present the methodology for OD localization and boundary segmentation. The results of our method are presented in Section 3. Comparison with results from same database by other methods can also be found in this section. Finally, Section 4 presented the conclusions of this study and future improvement.

## 2. Materials and Methods

The optic disc segmentation methodology of this paper can be divided into 3 main steps: (a) optic disc location, (b) vessel removal, and (c) optic nerve head segmentation.

### 2.1. Optic Disc Detection and ROI Selection

#### 2.1.1. Optic Disc Detection

**Figure 2:**
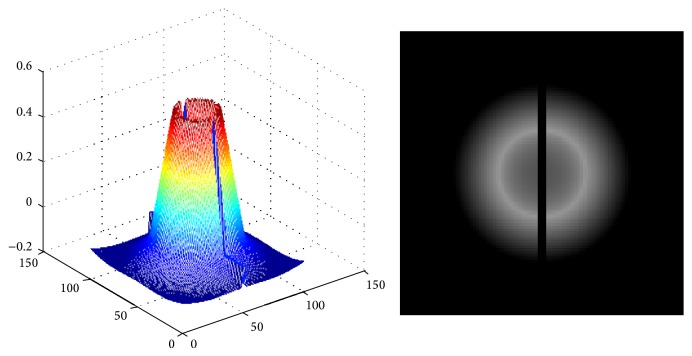
Optic disc template [12].

We use the template matching [9] to find the estimated disc region and then apply a circle Hough transform to correct the center and find an estimated disc radius. Since the appearance of the OD may vary significantly due to retinal pathologies, but the optic disc is the brightest feature of the normal fundus and shape of the optic disc is approximately vertically slightly oval (elliptical), a lot of templates have been proposed in [12] to find the optic disc. In this paper, we adopt the template from Lowell et al. [9] as shown in Figure 2. In our dataset the optic disc width is ranging from 260 to 380 pixels' length. So a template with size of 401∗401 is used in our experiments. Then the Pearson correlation coefficient is used to measure the correlation between the intensity channel from HSI color space subimage and the template in general:

(1) cij=∑x,yfx,y−fmtx−i,y−j−tm∑x,yfx,y−fm2∑x,ytx−i,y−j−tm2,

where *t*_*m*_ is the mean intensity values of the template, which need to be calculated only once. *f*_*m*_ is the mean intensity values of the image region covered by the template. The location with the maximum value is selected as the optic disc location. The method is robust to small exudate and can fast locate the optic disc. In the next step a circle Hough transform is used to update the center and find a disc radius.

#### 2.1.2. ROI Selection Based on Circle Hough Transform

**Figure 3:**
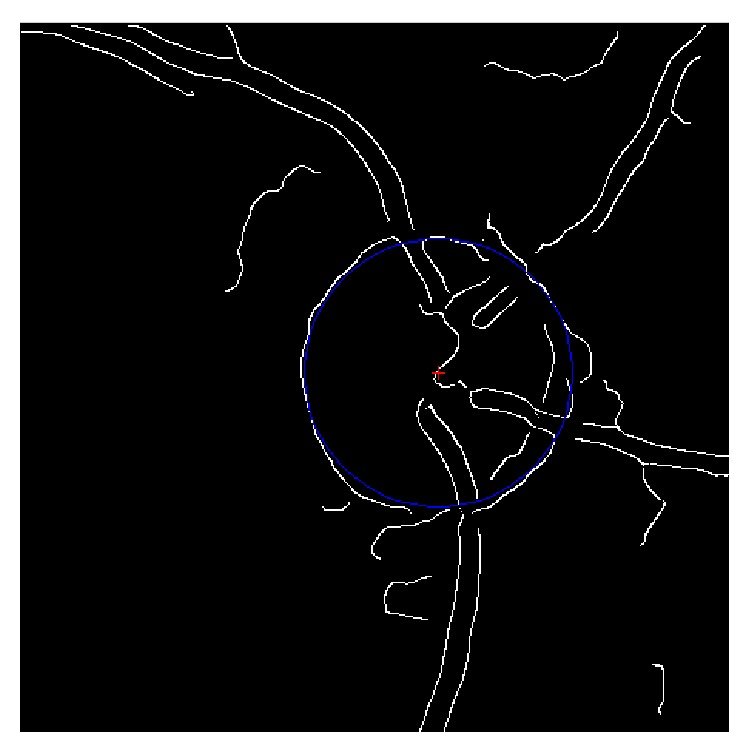
Result of circle Hough transform. The blue line is the estimated disc region.

Hough transform can be used to find the circular shape with fixed radius in the edge image of the fundus. An estimated disc center which is detected in the template matching step is selected as the region of interesting center. An appropriate region with size of 900∗900 is chosen as ROI. We smooth the image with a Gaussian filter to remove small noise. Canny kernel edge detection with a threshold value of 0.4 is applied to the ROI to remove weak boundaries and noises. Finally the Hough transform is applied to the edge pixels in the edge map to accumulate evidence of circles with fixed radius *R* in the image. In this paper, the radius is ranging from 140 to 230 with a step size of 15. The circle with the highest magnitude of evidence is chosen as the optic disc. The result is shown in Figure 3.

### 2.2. Vessel Removal

Large blood vessels extending from optic disc may influence the precise edge of the optic disc. A PDE based inpainting method [18] based on the vessel mask is adopted. We also try to use morphological closing operation and mean pixels replacement method. In the morphological closing approach, a disc with radius of 15 is chosen as the structuring element, while in the mean pixels replacement approach the vessel mask pixel value is replaced by the mean value of the neighbor region.

**Figure 4:**
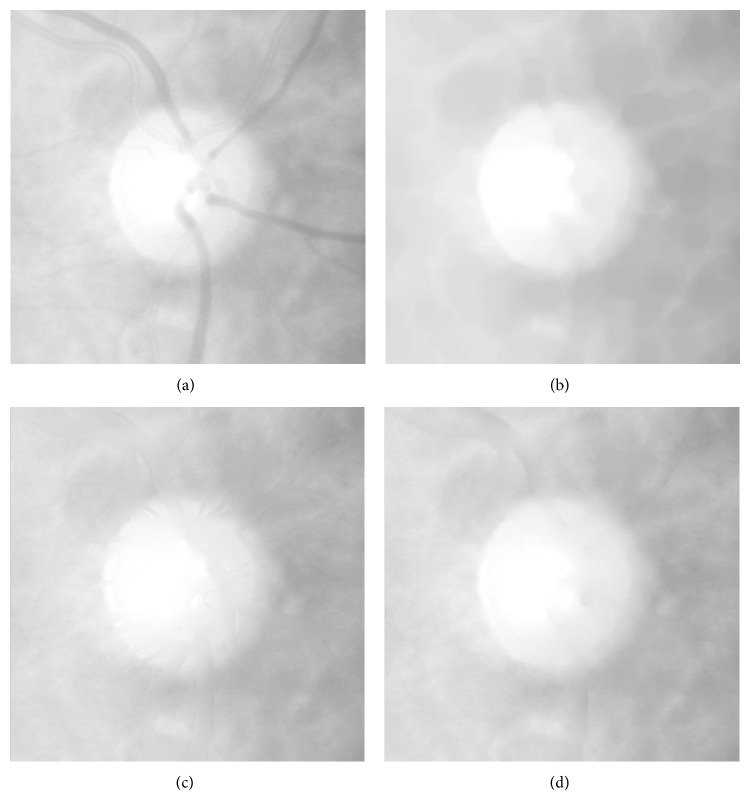
Vessel removal results. (a) Red channel image. (b) Close operation. (c) Mean pixels replacement. (d) PDE inpainting.

We apply above three vessel removal methods and the results are shown in Figure 4. The advantage of morphological closing operation is that you do not need to segment the vessel first but it destroys the image texture and makes optic disc boundary become blurred. The result of image inpainting is slightly better than pixels replacement method. Moreover, it is much faster than replacement method. After the vessel removal processing, there is little information about vessel left.

### 2.3. Optic Nerve Head Segmentation

#### 2.3.1. Image Texture

In order to improve the segmentation result of the optic disc, we need to find those features which can distinguish the optic disc region and boundary. Moreover, in consideration of the PPA region which surrounds the optic disc, we need to find those features that can also have different values between the optic disc and PPA region.

Schmid proposed texture feature filter banks which are rotationally invariant and are obtained by convolution with isotropic “Gabor-like” filters [19]. These filters combine different frequencies and scales together as follows:

(2) Fx,y,τ,σ =F0τ,σ+cos⁡⁡x2+y2πτσe−x2+y2/2σ2,

where *τ* is the number of cycles of the harmonic function within the Gaussian envelope of the filters. *σ* represents the scale of the filter.

**Figure 5:**
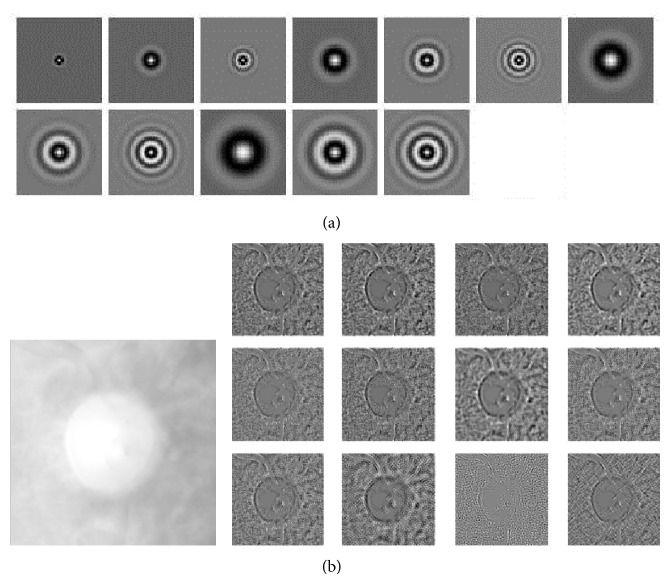
Schmid filter banks and results. (a) The Schmid filter bank. (b) The left one is the raw image. The right is the first twelve responses from Schmid.

In this paper, several filters are generated by taking different banks parameters (*σ*, *τ*) pairs. They are taken by ranges (2,1), (4,1), (4,2), (6,1), (6,2), (6,3), (8,1), (8,2), (8,3), (10,1), (10,2), and (10,3), 12 filters in all. The filters are shown in Figure 5(a). And the responses of the image from Schmid filter bank are shown in Figure 5(b).

**Figure 6:**
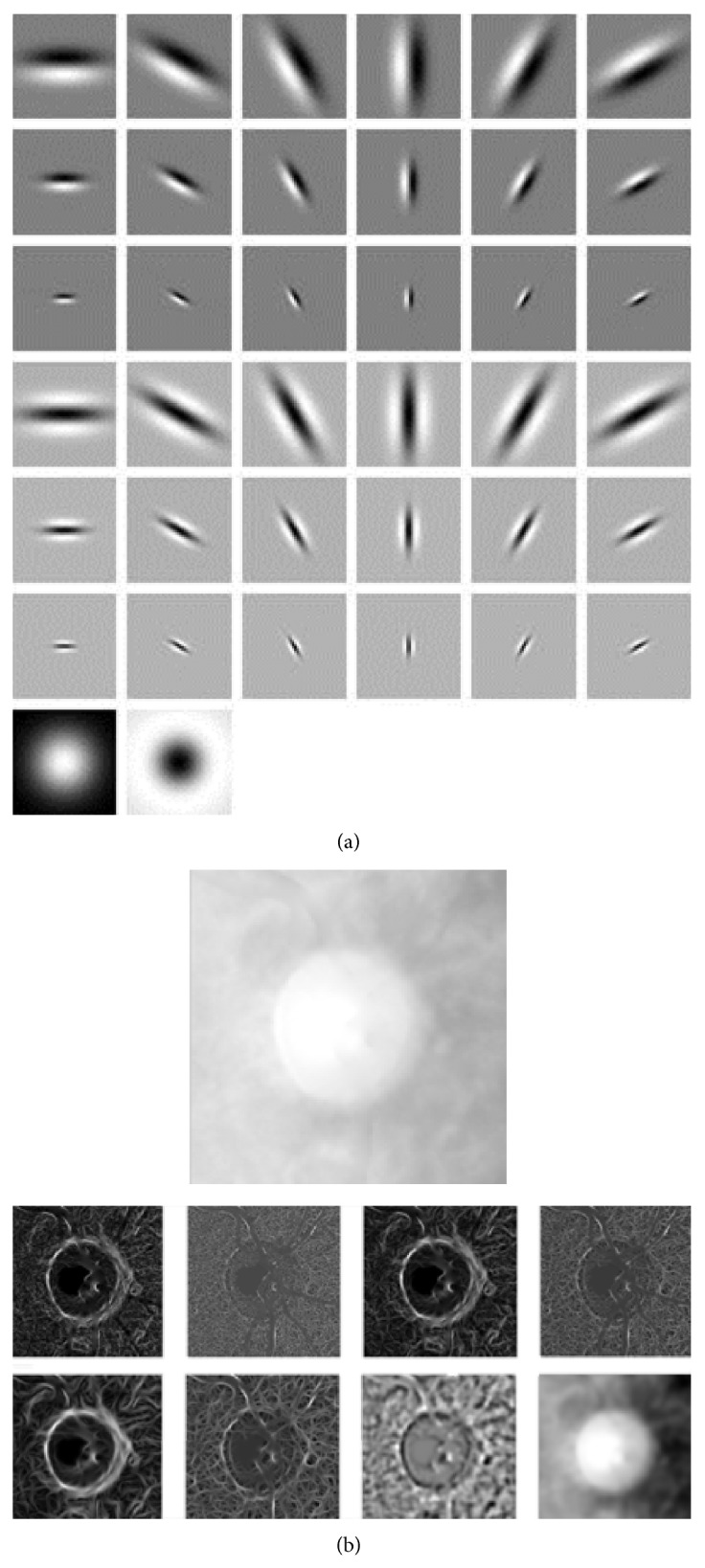
Root Filter Set and MR8 filter results. (a) Root Filter Set. (b) The upper part is the raw image. The lower part is the response result from MR8 filter.

Another filter bank adopted is the Maximum Response Filter Banks [20]. These filters are derived from a common Root Filter Set (RFS). The RFS consists of 38 filters and is very similar to the Leung-Malik filter bank [21]. It consists of first and second derivatives of Gaussians at 6 orientations and 3 scales, 36 filters in all, and the last two filters are a simple Gaussian and a Laplacian of Gaussian filter. To achieve rotational invariance, the authors get the Maximum Response Filter Bank from RFS by recording only the maximum filter response across all orientations for the two anisotropic filters [20]. By measuring only the maximum response across orientations, the author reduces the number of responses results from 38 to 8 (3 scales for 2 filters, plus 2 isotropic). The results of applying MR8 filter to the image are shown in Figure 6.

#### 2.3.2. Optic Disc Segmentation with Balloon Snake

*(1) The Balloon Snake.* Cohen [22] added a pressure term to make the model behave like an inflatable balloon or bubble that is trapped by strong edges but expands through edges that are weak relative to the pressure and smoothing forces. The energy functional of traditional snake is changed and a pressure force is added to the formulation:

(3) Eballoon=α2∮∂u∂s2ds︸Tension+β2∮∂2u∂s22ds︸Stiffness−ρ2∮∂u∂s×u ds︸Pressure+ξ∮PIuds︸Potential,

where the contour **u** depends on two parameters as below:

(4) $$\begin{array}{c} u\left(s,t\right)=\left(x\left(s,t\right),y\left(s,t\right)\right). \end{array}$$

**Figure 7:**
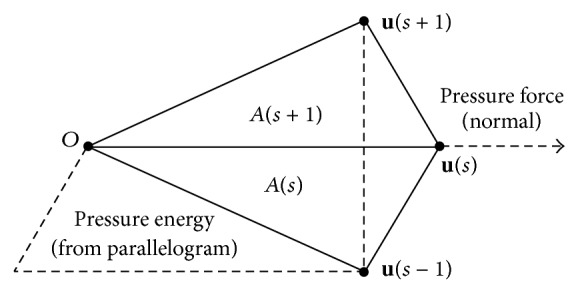
Pressure energy.

The third energy part in this equation is an isotropic pressure potential that controls the evolution of the area enclosed by the model. The energy of pressure is measured by size of the triangles region *A*_*s*_ in Figure 7.

The numerical solution of this equation can be found in [23]. At last we can get the total energy change of balloon snake as below:

(5) $$\begin{array}{c} \delta E=\oint \left(\frac{\partial P}{\partial u}-\alpha \frac{\partial^{2}u}{\partial s^{2}}+\beta \frac{\partial^{4}u}{\partial s^{4}}+\rho {\left(\frac{\partial u}{\partial s}\right)}^{⊥}\right)\cdot \delta u\;ds. \end{array}$$

At the limit of infinitesimal steps, the continuous descent equation is

(6) ∂u∂t=α∂2u∂s2︸Tension  Force−β∂4u∂s4︸Stiffness  Force−ρ∂u∂s⊥︸Pressure  Force−∂P∂u︸Image  Force.

**Figure 8:**
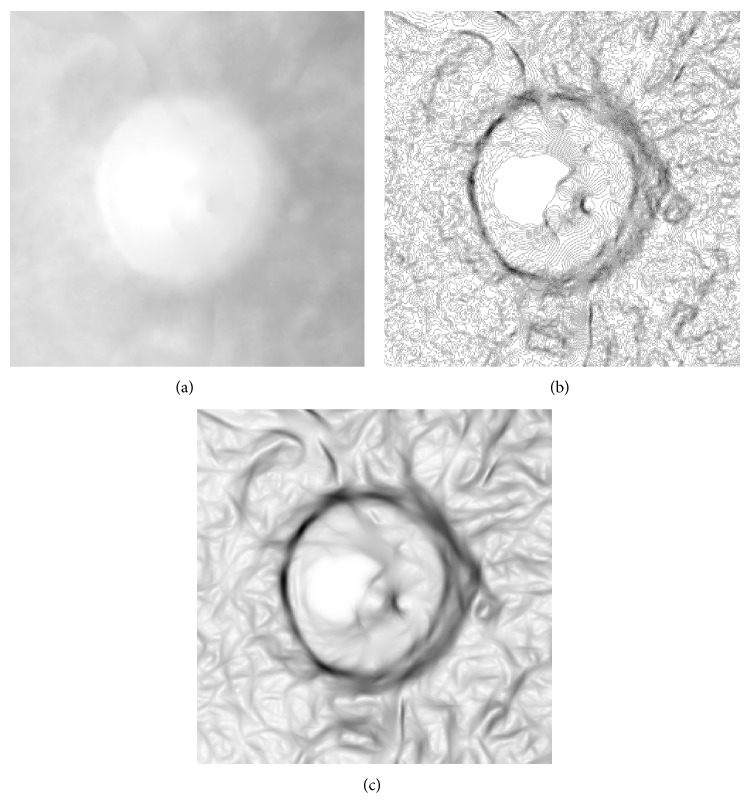
Image gradient and texture. (a) Raw image. (b) Image gradient. (c) 3rd response from MR8.

*(2) Texture Image Force.* Balloon snake is difficult to use when the edges of images are weak as the image force of balloon snake is still gradient. After lots of experiments we adopt the 3rd texture response from Maximum Response Filter Bank; see Figure 8. The third texture response is good for segmentation as the boundary information is clearer than gradient and the noise texture caused by optic cup and vessel is less notable.

So the image force of balloon model is changed from gradient to texture. The energy equation of image force is as below:

(7) $$\begin{array}{c} E_{\text{image}}\left(x,y\right)=-\text{MRFilter}⊙I\left(x,y\right), \end{array}$$

where MRFilter is the Maximum Response Filter Banks and ⊙ means convolution.

**Figure 9:**
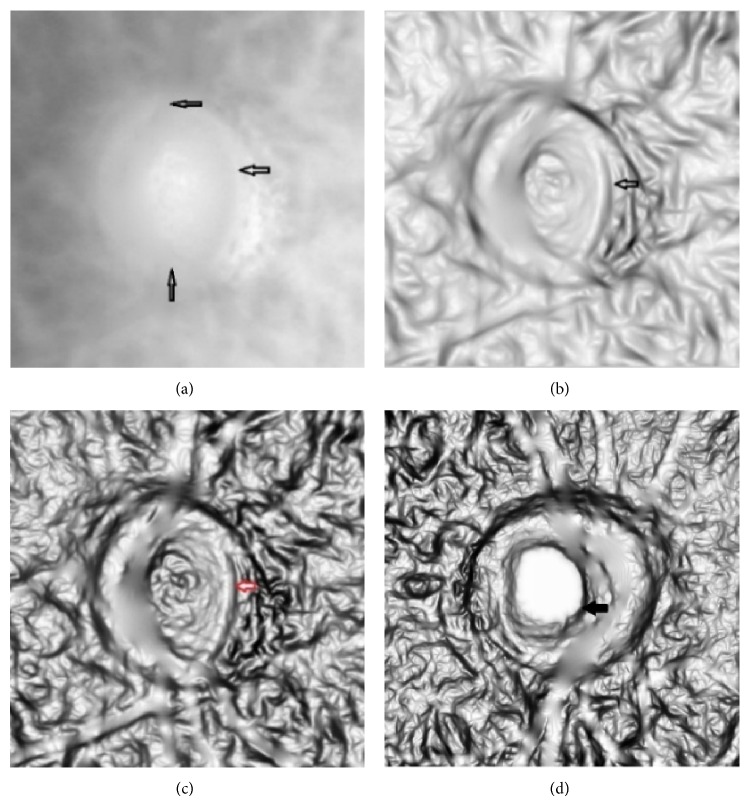
Image texture enhancement. (a) Raw image. The arrow indicates the week edges. (b) Image texture. The arrow indicates the fuzzy edge. (c) Texture enhancement. (d) Faked edges caused by enhancement.

*(3) Texture Enhancement.* Although this texture information is better than the image gradient for edge detection, there still remain some weak boundaries problems as shown in Figure 9(b). We adopt the contrast-limited adaptive histogram equalization (CLAHE) to enhance the edge features. The CLAHE operation is directly applied to the image texture rather than the raw ROI image, as the CLAHE is good at enhancing small objects such as edges, and the image texture is something like small objects. The result of texture enhancement is shown in Figure 9(c).

After CLAHE enhancement, the texture becomes clearer than before. However, the noise texture which is formed by cup boundary is also clearer as shown in Figure 9(d). This will reduce the segmentation result, as the initial contour is inside the optic disc and the balloon snake may stop at the false edges formed by cup boundary or the vessel in the nasal side.

*(4) Noise Textures Remove.* In order to remove all noises except the disc boundary, we need to exclude those noise features and retain the texture features near the boundary. In this paper, we proposed a clustering method to find the estimated edge region from the texture image by fuzzy *c* means approach. The detail of operation is as below.

*(a) ROI Selection.* According to estimated radius from the circle Hough transform, a larger estimated radius is selected to make sure the disc region is included in the new ROI. In this paper, we add the estimated radius by 50.

*(b) Feature Selection.* The feature space should have differences between the background and boundary or edge region. We give up the image intensity space as there is serious inhomogeneous intensity in fundus image which will make wrong classification. Three features are extracted from the red channel of ROI image. They are the 1st response from the Schmid filter bank and 5th and 7th response from Maximum Response (MR) Filter Bank. So every element point has three features; then they are classified into two clusters by fuzzy *c* means.

**Figure 10:**
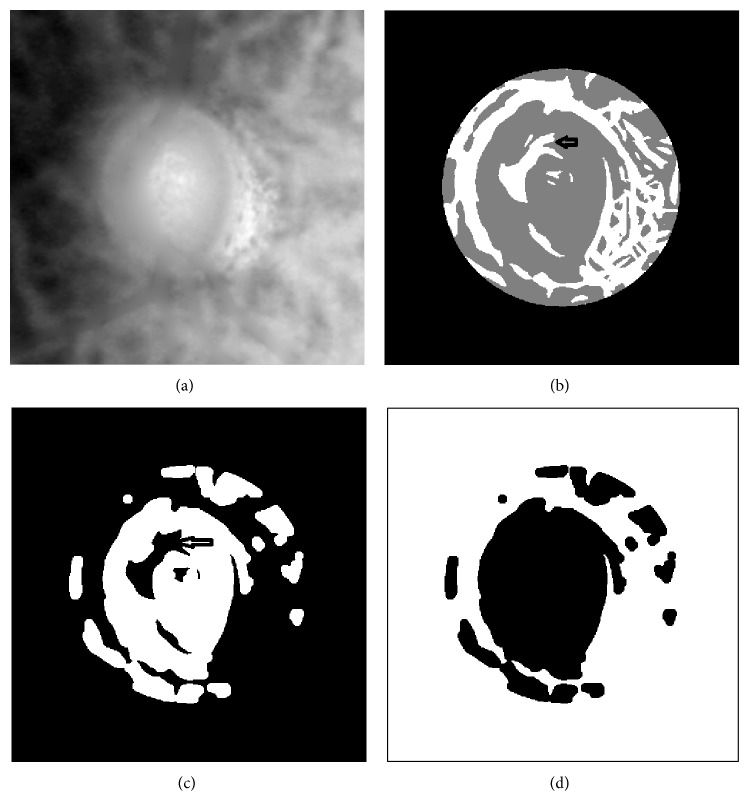
Edge region selection steps. (a) Raw image. (b) After fuzzy *c* means cluster. (c) After open operation. (d) Edge region.

*(c) Cluster Selection.* After the classifications, the region is divided into two groups as shown in Figure 10(b). The proposed knowledge-based clustering is based on the assumption that the background region is larger than estimated edge region. The max region is selected as the estimated background region, but there are still some holes in the background due to the cup boundary or the faked vessel edge. In order to remove the noise inside the background region, an open operation is applied to the background region as shown in Figure 10(c); this step will link the edge region; then we choose the opposite region, the estimated edge region, and select the max connect region as the estimated edge region. After this operation, small faked edge region is excluded from the edge region as shown in Figure 10(d).

**Figure 11:**
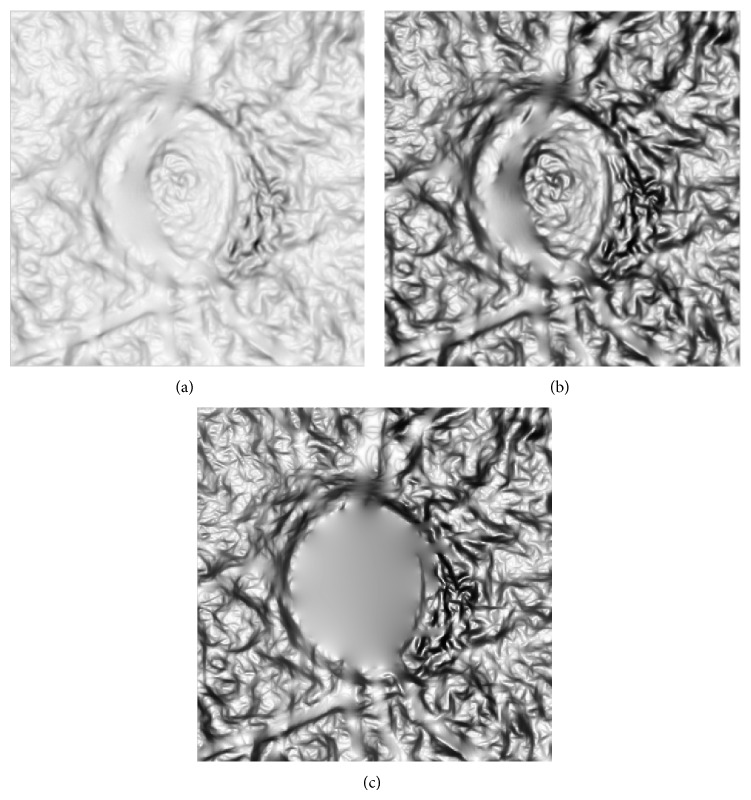
Texture noise removal. (a) Image texture. (b) After enhancement. (c) After noise inpainting.

*(d) Noise Inpainting.* After we get the edges mask, we need to remove those noises features in the optic disc. Based on the background mask from the preview step, we cannot simply assign 0 or 255 to the background region as it will produce a local maximum or minimum between background region and other regions which makes the snake contour stop. In order to make the contour smooth cross the background region, an inpainting approach [18] is employed to remove the texture noises. So in this step an inpainting operation is employed to the image again to remove the vessel edge and background region which is detected in the previous step. The final result of texture enhancement and texture noises removal is shown in Figure 11.

**Figure 12:**
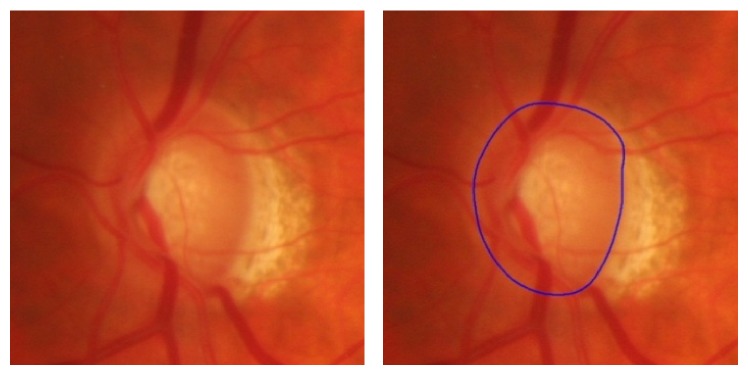
Balloon snake results.

*(e) The Deformable Balloon Contour.* In this paper, a circle with half of the estimated radius is adopted to make sure that the initial contour is inside the optic disc. The gradient energy is replaced by the texture energy. The contour is expanding like balloon and will stop at the texture boundary. The result of segmentation is shown in Figure 12. This segmentation method can avoid PPA as they are outside the optic disc. Moreover, if there is a PPA region near the disc, this contour will stop near the disc boundaries as long as there exists difference between the PPA and disc region.

## 3. Results and Discussion

### 3.1. Glaucoma Database

The database used in this paper is from High-Resolution Fundus (HRF) Image Database [24]. The database contains 15 images of healthy patients, 15 images of patients with diabetic retinopathy, and 15 images of glaucomatous patients at the moment. There are often some large exudates or bleeding in diabetic retinopathy images which make it difficult to locate the disc. In this paper, we choose 15 images of healthy patients and 15 images of glaucomatous patients from this database. Binary golden standard vessel segmentation images are available for each image. The manual optic disc mask which is used as golden truth was collected by one ophthalmology expert from He Eye hospital, Shenyang.

### 3.2. Performance of the Algorithm

In this paper, we adopt the dice coefficient to quantify the performance of the optic disc segmentation; the dice coefficient is defined as follows:

(8) $$\begin{array}{c} \text{DC}=\frac{2*\text{Area}\left(A\cap B\right)}{\text{Area}\left(A\right)+\text{Area}\left(B\right)}. \end{array}$$

*A* represents the ground truth and *B* represents our optic disc segmentation result. A DC value of 1 indicates a perfect segmentation result with the ground truth, and a small DC value indicates a bad segmentation.

**Figure 13:**
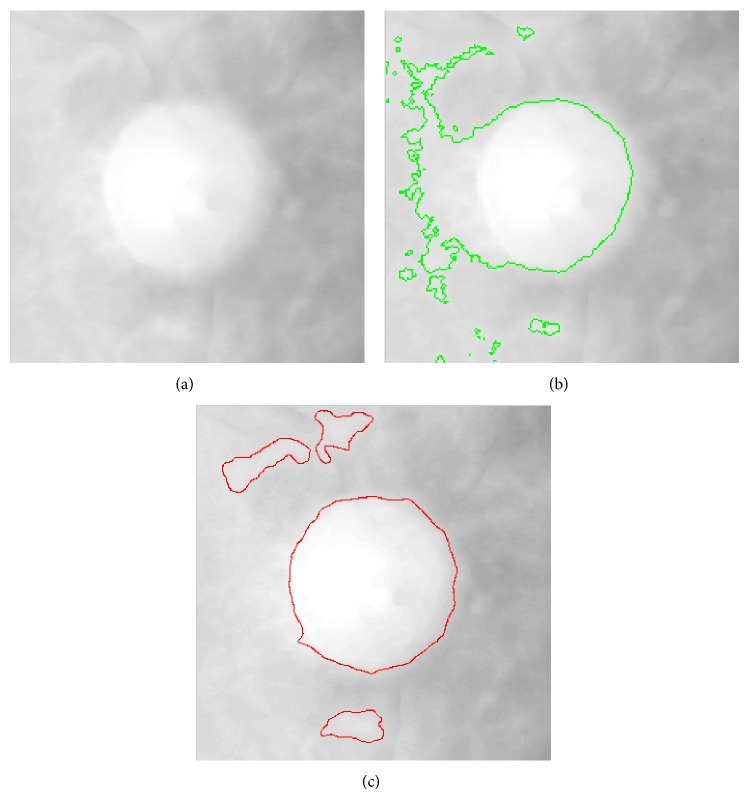
CV and local Gaussian distribution model. (a) Raw image. (b) CV model results. (c) Local Gaussian distribution model results.

Lots of approaches have been tested to segment the disc in our experiments. The Chan-Vese [25] (CV) and an improved approach, local Gaussian distribution model [15] (LGD), are state-of-the-art level set segmentation methods and they are employed to segment the optic disc. Although both of them are not sensitive to the initial contour, we make the initial contour be a circle with radius smaller than the estimated disc radius from the circle Hough transform. However, we find that the CV and LGD are not suitable to do disc segmentation as shown in Figure 13. In CV model, the image intensity is used as energy to find an edge which makes the sum of inner and external energy be the minimum. This method often fails in optic disc segmentation because of inhomogeneity as shown in Figure 13(b). This image has intensity inhomogeneity where the left part is brighter than the right part. So the CV model makes some bright region located in the left optic disc as a part of the optic disc.

While the LGD model is designed for handling image inhomogeneity problem, this method efficiently utilizes local image information rather than the global information such as CV model. We can see from Figure 13(c) that there are lots of small dark or bright regions near the optic disc which is caused by the light illumination or reflex. After we apply LGD to the ROI image, we find that it has the problem of oversegmentation as the energy minimization is achieved by an interleaved level set evolution and estimation of local intensity means and variances in an iterative process. This approach will falsely segment some small regions. So we give up these two methods and regard the edge based active contour model.

We compare our proposed method with traditional snake and another texture cluster methods which is fuzzy *c* means cluster method. And in the texture cluster segmentation method, we adopt the 5th response from MR8 and 1st response from Schmid filter bank of the red channel values of image, 2 features for the FCM input. Then we apply morphological operation to the larger region, and an ellipse fit is applied to get this region boundary.

**Figure 14:**
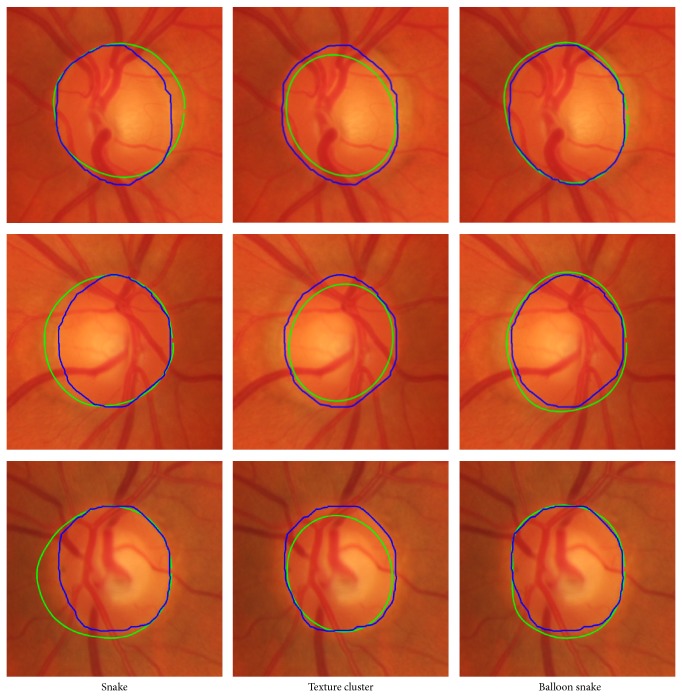
Three segmentation approaches. The blue is the golden mask and green is the segmentation result.

**Figure 15:**
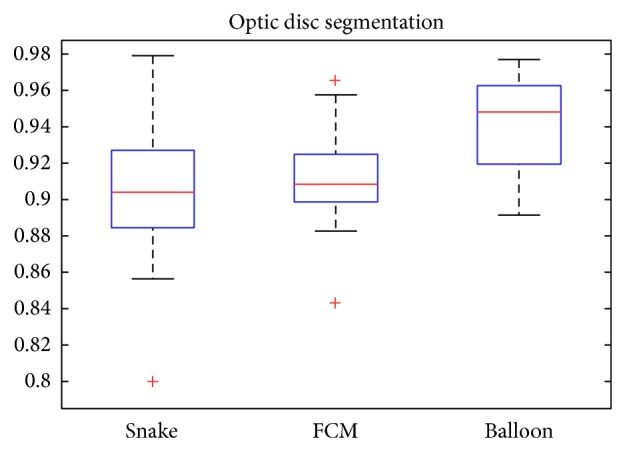
Optic disc segmentation results over 30 images.

As shown in Figure 14, we can see from the image that the snake model often fails to detect the true edge of disc because of the fuzzy edge and PPA region. The results often include the PPA region as a part of disc. To sum up, our proposed segmentation method is more robust to the snake, as you can see from Figure 15. In the box-and-whisker figure, the proposed balloon snake is better than other two approaches, and most of segmentation dice coefficient is 94% as shown in Table 1. Although the mean dice coefficient of FCM texture cluster approach is almost the same as snake model, most of coefficient is distributed more than 90%. We utilize the Matlab (MathWorks, Inc., Natick, MA) and Mathematica (Wolfram Research, Champaign, IL) to implement our method mentioned in this paper. It costs about 2 minutes to process one image on PC (I5 Intel CPU and 4 G RAM).

## 4. Conclusions

In this paper, we focus on automatic segmentation of the optic disc based on 2D fundus image. Template matching and circle Hough transform are used to locate the optic disc and find an estimated circle radius. In the vessel erasing step, we use inpainting approach to remove vessel which is better than the closing operation and mean neighbor replacement.

Our proposed optic disc segmentation method is based on image texture and active contour model. We use the balloon snake model which makes the contour expand like balloon from the inner optic disc. This method can avoid the PPA region outside the disc boundaries and is less sensitive to the initial contour, as the initial contour of normal snake model and GVF model are recommended near the true disc boundaries. The main problem of this balloon model is the texture noise which is caused by faked vessel edges, and sometime the cup is obvious in the red channel which will make this contour mistake the cup boundary as the disc edge. So we proposed a cluster based approach to remove those texture noises in the disc. The results from our proposed approach are better than the snake model and fuzzy *c* means cluster approach.

The optic nerve head segmentation results show that the proposed method is able to achieve better segmentation accuracy for optic disc comparing to other existing methods. The works of this paper as a whole can be used in retina image processing system, which is highly desirable for large-scale screening programs. However, our proposed method is sensitive to optic disc location approach and vessel segmentation results; in the future we need to adopt a better optic disc location method which can handle large exudate in the diabetic retinopathy images and make our method more robust to handle inaccurate vessel segmentation results.

## Figures and Tables

**Table 1:** Disc segmentation results.

	*μ*	*δ*
Snake	0.9053	0.0445
Texture cluster	0.9093	0.0311
Our method	0.9400	0.0263
